# Prognostic Significance of Interleukin-8 and CD163-Positive Cell-Infiltration in Tumor Tissues in Patients with Oral Squamous Cell Carcinoma

**DOI:** 10.1371/journal.pone.0110378

**Published:** 2014-12-02

**Authors:** Yohei Fujita, Masato Okamoto, Hiroyuki Goda, Tomoyuki Tano, Koh-ichi Nakashiro, Atsuro Sugita, Tomonobu Fujita, Shigeo Koido, Sadamu Homma, Yutaka Kawakami, Hiroyuki Hamakawa

**Affiliations:** 1 Department of Oral and Maxillofacial Surgery, Ehime University Graduate School of Medicine, Ehime, Japan; 2 Department of Advanced Immunotherapeutics, Kitasato University School of Pharmacy, Tokyo, Japan; 3 Division of Cellular Signaling, Institute for Advanced Medical Research, Keio University School of Medicine, Tokyo, Japan; 4 Department of Pathology, Ehime University Hospital, Ehime, Japan; 5 Division of Gastroenterology and Hepatology, Department of Internal Medicine, The Jikei University School of Medicine, Tokyo, Japan; 6 Department of Oncology, Institute of DNA Medicine, The Jikei University School of Medicine, Tokyo, Japan; Istituto dei tumori Fondazione Pascale, Italy

## Abstract

**Purpose:**

We investigated whether serum interleukin (IL)-8 reflects the tumor microenvironment and has prognostic value in patients with oral squamous cell carcinoma (OSCC).

**Experimental Design:**

Fifty OSCC patients who received radical resection of their tumor(s) were enrolled. Preoperative sera were measured for IL-8 by ELISA. Expression of IL-8 and the infiltration of immune cells in tumor tissues were analyzed by an immunohistochemical staining of surgical specimens.

**Results:**

We found that disease-free survival (DFS) was significantly longer in the Stage I/II OSCC patients with low serum IL-8 levels compared to those with high levels (p = 0.001). The tumor expression of IL-8, i.e., IL-8(T) and the density of CD163-positive cells in the tumor invasive front, i.e., CD163(IF) were correlated with the serum IL-8 level (p = 0.033 and p = 0.038, respectively), and they were associated with poor clinical outcome (p = 0.007 and p = 0.002, respectively, in DFS) in all patients. A multivariate analysis revealed that N status, IL-8(T) and CD163(IF) significantly affected the DFS of the patients. Further analysis suggested that combination of N status with serum IL-8, IL-8(T) or CD163(IF) may be a new criterion for discriminating between OSCC patients at high and low risk for tumor relapse. Interestingly, the in vitro experiments demonstrated that IL-8 enhanced generation of CD163-positive M2 macrophages from peripheral blood monocytes, and that the cells produced IL-10.

**Conclusions:**

These findings indicate that IL-8 may be involved in poor clinical outcomes via generation of CD163-positive M2 macrophages, and that these factors in addition to N status may have prognostic value in patients with resectable OSCSS.

## Introduction

Head and neck squamous cell carcinoma (HNSCC) represents the fifth most frequently occurring cancer worldwide. Of the 1.6 million diagnoses and 333,000 deaths each year worldwide due to HNSCC, one-half are localized in the oral cavity [oral squamous cell carcinoma (OSCC)] [1]. Despite recent advances in surgery, radiotherapy and chemotherapy, the 5-year survival rate for patients with OSCC has remained at 50% for the past 30 years [2]. The treatment for patients with early-stage OSCC (Stage I or II) as well as for those with advanced OSCC (Stage III or IV) is mainly surgical resection. The desired improvement in the efficacy of treatment for OSCC will be aided by the identification of biomarker(s) that can identify the subpopulation of OSCC patients who are at high risk of tumor relapse, and by the development of effective treatments for these high-risk patients. Although Tumor-Node-Metastasis (TMN) classification-based staging is an important prognostic factor in OSCC patients, the prognosis is not satisfactory even in early-stage patients, and high-risk patients who are Stage I/II OSCC might be missed based on the TNM staging [3]–[6].

We have reported the prognostic significance of the expression ratio of the genes for the anti-apoptotic protein Bcl-2 and the pro-apoptotic protein Bax in circulating immune cells, and we found that the immunological status might be critical to the clinical outcome of patients with head and neck cancer [7]. However, the usefulness of this information is not yet confirmed because it is still unknown what tumor microenvironment is reflected by this immunological condition of the peripheral blood. The immune status in a tumor microenvironment is closely associated with the clinical outcomes of patients with malignancies [8.9]. The migration of the T cells positive for CD3, CD8 or FOXP3 into the tumor sites was reported to be correlated with the outcome of patients with several types of malignancies (i.e., ovarian, colorectal, and breast cancer as well as head and neck cancer) [8.9]. If a patient’s peripheral blood profile reflects the microenvironment of his or her tumor, it may be possible to estimate the immune status of the tumor microenvironment and to predict the patient’s clinical outcome by evaluating the immunological state of the peripheral blood. In fact, a recent study demonstrated that pre-therapeutic plasma interleukin (IL)-6 levels were correlated with the expression of nuclear factor (NF)-κB in the nuclei of tumor cells as well as the expression of IL-6 in local tumor sites, and that the plasma IL-6 level is an independent negative prognostic factor for overall survival (OS) of patients with castration-resistant prostatic carcinoma [10]. Motomura et al. reported that the pre-operative neutrophil-lymphocyte ratio (NLR) reflects hepatocellular carcinoma (HCC) recurrence after liver transplantation via an inflammatory tumor microenvironment [11]. Serum factor(s) that reflect the immune status of the tumor microenvironment may be useful prognostic biomarker(s).

We previously examined sera derived from OSCC patients for multiple cytokines by using a multiplexed measurement system [12], and the results showed that the serum IL-8 level tended to negatively correlate with favorable outcome in these patients (authors’ personal communications). In the present study, we expanded the number of patients with resectable OSCC, measured their sera for circulating IL-8 by an enzyme-linked immunosorbent assay (ELISA) which is more quantitative than the multiplexed measurement system, and compared the serum IL-8 levels with the clinical outcomes of the patients. In addition, to determine whether circulating IL-8 levels reflect the tumor microenvironment, especially the immunological microenvironment, we used immunohistochemical staining to analyze the expression of IL-8 as well as the infiltration of immune-inhibitory cells such as Foxp3-positeve regulatory T cells (Tregs) and CD163-positive M2 macrophages, which may be adverse prognostic markers [8], [9], [13].

## Materials and Methods

### Patients

This study was carried out in accord with the standards of our Institutional Committee for the Protection of Human Subjects. We have read the Helsinki Declaration and have followed the guidelines in this investigation. Informed written consent was obtained from all patients, and the collection of the samples was approved by the Institutional Review Board of Ehime University Hospital [IRB approval No. 1308006 (Title: A search of factors influencing the decrease of host immunity in patients with oral squamous cell carcinoma and No. 1309015 (Title: An analysis for the mechanism of the inhibition of host immunity in patients with oral malignancies)]. From April 2006 to March 2010, 50 OSCC patients (32 males and 18 females) who received radical resection of their tumor(s) at the Division of Oral and Maxillofacial Surgery, Ehime University Hospital were enrolled in this study. A summary of the patients’ profiles is given in Table 1. The histological differentiation of the tumors was classified in accord with the WHO classification. The mode of cancer invasion was classified into five grades according to the classification proposed by Yamamoto and Kohama (YK classification): YK-1, well-defined border; YK-2, cords, less marked border; YK-3, groups of cells, no distinct border; YK-4C; diffuse invasion, cord-like type; and YK4-D, diffuse invasion, widespread type [14]. To evaluate the nutritional conditions of the OSCC patients, we used the Prognostic Nutritional Index (PNI = 10×serum albumin (g/dl) +0.005×total lymphocytes counts (/mm^3^)) [15].

**Table 1:** The clinicopathological characteristics of the OSCC patients.

			*p* value			
			Serum IL-8	IL-8 (T)	CD163 (IF)	
Numbers ofpatients		50				
Age		68.6 (range: 48–93)	0.557	1.000	1.000	
Sex	Male	32 (64%)	0.768	0.764	0.769	
	Female	18 (36%)				
Stage	I	9 (18%)	0.077	0.773	1.000	Stage I/II vs Stage III/IV
	II	18 (26%)				
	III	4 (8%)				
	IV	19 (38%)				
T	1	10 (20%)	0.199	1.000	0.127	T1/2 vs T3/4
	2	25 (50%)				
	3	3 (6%)				
	4	12 (24%)				
N	0	32 (64%)	0.540	0.130	0.377	
	1–3	18 (36%)				
M	0	50 (100%)	NA	NA	NA	
	1	0 (0%)				
Histologicaldifferentiation	well	40 (80%)	1.000	0.722	0.074	well vs non-well
	moderate	8 (16%)				
	poor	2 (4%)				
Mode of cancerinvasion	YK-1	0 (0%)	0.494	0.175	0.171	YK-1,2,3 vs YK-4
	YK-2	5 (10%)				
	YK-3	34 (68%)				
	YK-4C	10 (20%)				
	YK-4D	1 (2%)				
Primary sites	Tonge	17 (34%)	0.748	0.802	1.000	Tongue vs gingiva
	Upper gingiva	7 (14%)				
	lower gingiva	16 (32%)				
	Buccal mucosa	6 (12%)				
	Floor of the mouth	3 (6%)				
	mandiblar bone	1 (2%)				

The mode of cancer invasion was classified into five grades according to the classification proposed by Yamamoto and Kohama (YK classification).

NA: not analyzed.

### Serum collection and IL-8 ELISA

Before the patients’ surgery, their sera were collected and immediately frozen at −80°C until the assay for IL-8. We analyzed the serum IL-8 levels using a human CXCR8/IL-8 Quantikine ELISA kit (R&D Systems, Minneapolis, MN, USA) according to the manufacturer’s protocol. The cut-off value 7.5 pg/ml was chosen based on the sensitivity of the ELISA Kit and the receiver operating characteristic (ROC) curve. The serum IL-8 levels of healthy donors [5 males and 3 females, mean of ages 59.2 (range: 48–70)] were undetectable. Informed written consent was also obtained from all healthy donors.

### Immunohistochemical staining

The surgically resected OSCC specimens were fixed in phosphate-buffered 10% formalin and embedded in paraffin, and then a series of tissue sections (4 µm thick) were prepared from each sample. Immunohistochemical staining was performed by the avidin-biotin-peroxidase complex method. Briefly, the sections were deparaffinized, pretreated with 10 mM citrate buffer (pH 6.0) in an autoclave at 121°C for 20 minutes, and incubated with 0.3% H_2_O_2_ in distilled water for 10 minutes to block endogenous peroxidase activity. The sections were then incubated overnight at 4°C with each specific monoclonal antibody to human IL-8 (diluted 1∶50, DAKO, Glostrup, Denmark), to human Foxp3 (diluted 1∶100, eBioscience, San Diego, CA), and to human CD163 (diluted 1∶200, Novocastra, Newcastle, UK). After washing, the sections were overlaid with biotinylated anti-mouse antibody (Vector Laboratories, Burlingame, CA) at room temperature for 60 minutes and then washed in phosphate-buffered saline (PBS), followed by labeling with streptavidin-peroxidase complex (Vector Laboratories). The peroxidase reaction was developed with 3′3-diaminobenzidine as a chromogen. The sections were counterstained with hematoxylin, dehydrated with ethanol, treated with xylene and enclosed in synthetic resin. Quantitative studies of the immunohistochemically stained sections were performed by pathologists in a blind fashion by evaluating three randomly chosen fields in each sample. Individual cells were counted under microscopic fields. The cells stained with anti-IL-8 antibody were counted on tumor cells (IL-8(T)) and on stromal cells (IL-8(S)) in tumor tissues, and the cases in which over 5% of the cells were stained, were defined as positive expression (+). We counted the numbers of Foxp3- or CD163-stained immune cells that had infiltrated into the tumor (Foxp3(IT) or CD163(IT)) and those that had infiltrated the tumor invasive front (Foxp3(IF) or CD163(IF)) by using BIOREVO BZ-9000 (Keyence, Elmwood Park, NJ), and each median value was chosen as the cut-off value.

### Generation of CD163-positive M2 macrophages in vitro and the flow cytometric analysis of cell surface CD163 expression

The induction of CD163-positive M2 macrophages in vitro was carried out following the method of Durafurt et al [16]. In brief, healthy donor-derived PBMCs (5×10^6^/ml) prepared by the standard Ficoll-Hypaque gradient density centrifugation method were placed in 100-mm plastic tissue culture dishes (Becton Dickinson Labware, Franklin Lakes, NJ) in RPMI 1640 medium supplemented with 10% heat-inactivated FBS. After 3 hours of incubation at 37°C, nonadherent cells were removed, and the adherent cells were treated with recombinant human macrophage-colony stimulating factor (M-CSF) (25 ng/ml; PeproTech, Rocky Hill, NJ) for 5 days, and then were stimulated with recombinant human IL-4 (20 ng/ml, Invitrogen, Carlsbad, CA) and recombinant human IL-13 (20 ng/ml, PeproTech) for 2 days. We found previously by a flow cytometric analysis that the population of adherent cells remaining in the wells was composed of >95% CD14^+^ monocytes [17]. To examine whether IL-8 enhance the generation of M2 macrophages, 10 ng/ml of recombinant human IL-8 (R&D Systems) instead of IL-4 and IL-13 was added into the culture after 5 days of cultivation with M-CSF. We have already examined the dose dependency of IL-8 in IL-10 production in the preliminary study. IL-10 production showed the peal level in 10 ng/ml of IL-8 among the several concentrations (100 pg/ml, 1 ng/ml, 10 ng/ml and 100 ng/ml), and therefore 10 ng/ml of IL-8 was used in the current experiments. After 2 days, we evaluated the cell-surface expression of CD163 using a flow cytometric analysis, and measured the culture supernatants for IL-10 by ELISA (R&D Systems). Monoclonal antibodies for CD163 as well as for CD206 which is another marker of M2 macrophage and an isotype-matched control mouse IgG conjugated with fluoresceine-isothiocyanate (FITC) were purchased from PharMingen (San Diego, CA). The cells were resuspended in PBS containing 0.1% sodium azide and 0.2% bovine serum albumin and were then incubated for 30 minutes at 4°C with a saturating concentration of each monoclonal antibody according to the manufacturer’s instructions. After the cells were washed twice, their fluorescence intensity was determined using a flow cytometer (EPICS XL-MCL, Beckman Coulter, Fullerton, CA).

### Statistical analysis

We used Kaplan-Meier curves and log-rank tests to assess the differences in survival times between the treatment groups. A multivariate analysis was performed to evaluate the impact of factors in survival using Cox’s proportional hazards regression model. The differences between the two groups of categorical data were analyzed by two-sided Fisher’s exact test. In the *in vitro* experiments, we evaluated the data using Student’s two-tailed *t*-test. Values of *P*<0.05 were considered significant.

## Results

### Relationship between the clinicopathological characteristics of OSCC patients and serum IL-8 levels, IL-8 expression in the tumors and CD163-positive cell infiltration into the tumor invasive front

The immunohistochemical staining showed IL-8 expression in the tumor cells (IL-8(T)); most of the IL-8 expression in tumor tissues was observed in tumor cells but not tumor stromal cells. Very weak staining of IL-8 was observed in tumor stromal cells in 11 of 50 cases. However, no significant relationship to clinical outcome (DFS and OS) was observed (data not shown). Since the infiltration of CD163-positive cells into the tumor invasive front (CD163(IF)) but not the intra-tumor site (CD163(IT)) was strongly correlated with clinical outcomes (Overall survival [OS] and disease-free survival [DFS]) of the OSCC patients as described below, we present only the data of CD163 (IF) herein. In addition, the migration of Foxp3-positive cells into the tumor tissues was not significantly correlated with the OS as well as DFS (data not shown), and thus the data obtained with Foxp3-staining are not shown in this report.

We found no significant relationship between age, gender, TNM classification, histological differentiation, mode of cancer invasion, or primary site with serum IL-8, IL-8(T) and CD163(IF). The serum IL-8 levels tended to be high in the 23 patients with advanced-stage (Stage III/IV) of OSCC compared to the 27 early-stage (Stage I/II) patients, although no significant relationship was observed (*P* = 0.077) (Table 1).

### Relationship between the OS and DFS of OSCC patients and serum IL-8, IL-8 expression in tumor, and CD163-positive cell-infiltration into the tumor invasive front

**Figure 1:**
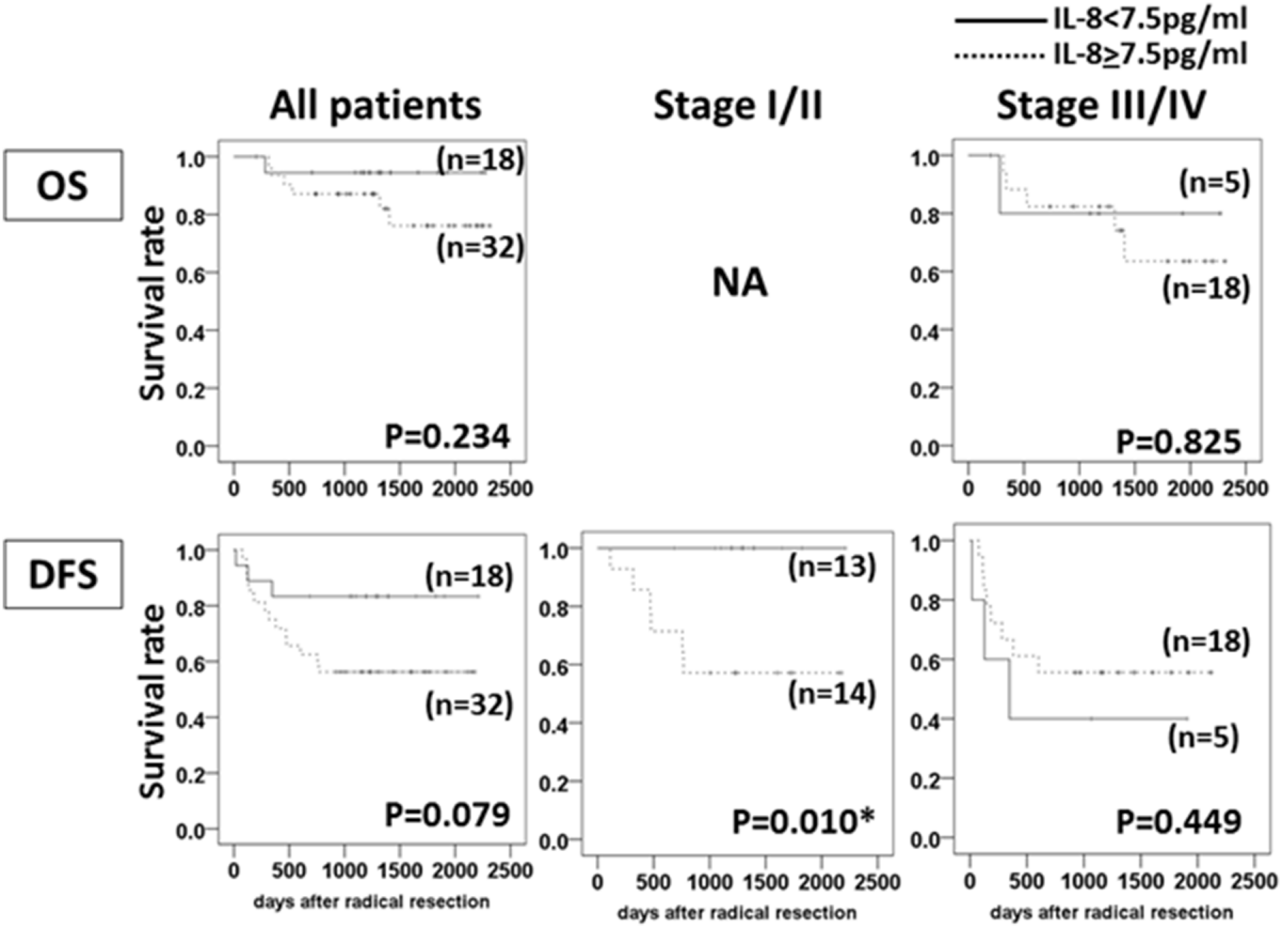
The relationship between serum IL-8 levels and the clinical outcome in OSCC patients who underwent radical resection of their tumor(s). The differences in the serum IL-8≥7.5 pg/ml vs. IL-8<7.0 pg/ml in all patients, in the Stage I/II patients and in the Stage III/IV patients were calculated by log-rank test. *P<0.05. NA: not analyzed because the number of OS events was only one.

Among the 50 OSCC patients, the 18 patients with low levels of serum IL-8 tended to be long survivors (*P* = 0.234 in OS, *P* = 0.079 in DFS), but a significant difference was not observed. Among the 27 Stage I/II patients, the DFS of the patients with low serum IL-8 levels were significantly longer than those of the patients with high serum IL-8 (*P* = 0.010), and in all of the Stage I/II patients with low IL-8, there have been no relapse events as of this writing. Among the 23 Stage III/IV patients, no significant relationship between the serum IL-8 level and clinical outcome (*P* = 0.825 in OS, *P* = 0.449 in DFS) was observed, as most of the patients with advanced OSCC (18 of the 23 patients) showed high levels of IL-8 in their sera (Fig. 1).

**Figure 2:**
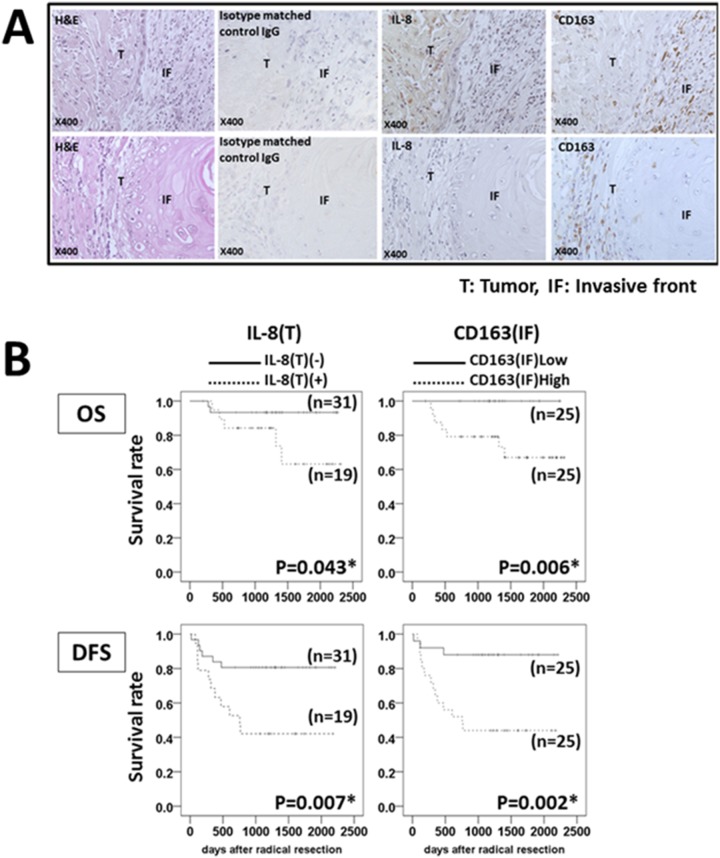
Relationship between IL-8 expression, CD163-positive cell-infiltration in tumor tissues and clinical outcome of OSCC patients. (A) Immunohistochemical staining for IL-8 and CD163. Most of the IL-8-positive cells were tumor cells, not stromal cells in tumor tissues. CD163-positive cells had infiltrated into the tumor invasive front but not the intra-tumor region. (B) Differences in IL-8(T)(+) vs. IL-8(T)(−) and CD163(IF)High vs. CD163(IF)Low in OS and DFS in OSCC patients, calculated by log-rank test. *P<0.05.

We examined the expression of IL-8 in tumor cells (IL-8(T)) and CD163-positive cell-infiltration into the tumor invasive front (CD163(IF)) by immunohistochemical staining. Upper panel of Fig. 2A demonstrated a case who showed IL-8(T)(+) and CD163(IF)High. Fig. 2A lower panel showed a rare case who showed IL-8(T)(−) and CD163(IF)High. Any factor(s) except for IL-8 might play a role for migration of CD163-positive cells. We analyzed their relationships with the patients’ clinical outcome. In 16 of the 19 IL-8(T)(+) cases, CD163-positive cells migrated to the tumor invasive front in proximity to cancer cells expressing IL-8 (Fig. 2A, upper panel). The OS and DFS of the IL-8(T)(−) patients were significantly longer than those of the IL-8(T)(+) patients (*P* = 0.043 in OS, *P* = 0.007 in DFS). The patients with low CD163(IF) showed longer OS and DFS compared to the patients with high CD163(IF) (*P* = 0.006 in OS, *P* = 0.002 in DFS) (Fig. 2B). In these cases, 15 of 16 IL-8T(+)CD163(IF)High patients showed high serum IL-8, and only 1 case low serum IL-8 (data not shown).

### Correlation of the mode of relapse with serum IL-8, IL-8(T) and CD163(IF)

Serum IL-8, IL-8(T) and CD163(IF) were each significantly correlated with post-operative cervical lymph node (LN) metastasis (*P* = 0.018, *P* = 0.001 and *P* = 0.023, respectively). Although no significant relationship was observed, IL-8(T) and CD163(IF) also tended to be correlated with local recurrence (*P* = 0.089 and *P* = 0.098, respectively) (Table 2). Only T status was significantly related to local recurrence (*p* = 0.002) (Table 2); the frequency of post-operative LN metastasis was not correlated with T status (Table 2) or with the mode of invasion (data not shown).

**Table 2:** Relationship between mode of relapse and IL-8, CD163+cell-infiltration.

	Local recurrence	Post-operative LN metastasis
	(*p*-value)	
Serum IL-8	1.000	0.018*
IL-8(T)	0.089	0.001*
CD163(IF)	0.098	0.023*
T status	0.002*	0.423

H: high, L: low. LN: lymph node. The statistical relationships were detected by two-sided Fisher exact test.

*denotes *p*<0.05, indicating statistically significance.

### Correlations among serum IL-8, IL-8(T) and CD163(IF)

In all patients, a significant correlation was observed between serum IL-8 levels and IL-8(T) (*p* = 0.033), and this correlation was much stronger in the early-stage OSCC patients (*p* = 0.018). No such correlation was observed in the patients with advanced OSCC (*p* = 0.621) (Table 3). A similar correlation or tendency was observed between serum IL-8 and CD163(IF) (*p* = 0.038 in all patients, *p* = 0.021 in Stage I/II patients, and *p* = 0.640 in Stage III/IV patients) (Table 3). A marked and significant relationship between IL-8(T) and CD163(IF) was observed in all 50 patients (*P* = 0.0003) (Table 3).

**Table 3:** Relationship between serum IL-8 and IL-8(T), between serum IL-8 and CD163(IF), between IL-8(T) and CD163(IF).

	All patients	Stage I/II	Stage III/IV
	(*p*-value)		
Serum IL-8 vs. IL-8(T)	0.033*	0.018*	0.621
Serum IL-8 vs. CD163(IF)	0.038*	0.021*	0.640
IL-8 (T) vs. CD163 (IF)	0.0003*	0.0003*	0.193

The statistical relationships were detected by two-sided Fisher exact test. * denotes P<0.05, indicating statistically significance.

We analyzed the relationship between the expression of IL-8 in tumor cells, i.e., IL-8(T) and the patients’ peripheral blood data and found that there was a significant correction between IL-8(T) and the circulating C-reactive protein (CRP) level (*P* = 0.001) (Data not shown). The serum IL-8 level was correlated with CRP only in the Stage I/II OSCC patients (data not shown).

### Univariate and multivariate analyses of factors affecting DFS from surgical resection

T status, IL-8(T) and CD163(IF) were significantly correlated with DFS (*P* = 0.028, *P* = 0.007 and *P* = 0.002, respectively), and the N status, CRP, PNI and serum IL-8 tended to be correlated with DFS (*P* = 0.139, *P* = 0.080, *P* = 0.099 and *P* = 0.079, respectively) by log-rank test (Table 4). Although high serum albumin level also tended to be correlated with long DFS, PNI showed a higher p-value, and thus we show the PNI data in Table 4.

**Table 4:** Univariate and multivariate analyses of factors affecting DFS after surgical resection of OSCC.

Variables		No. of patients	Log-rank (p-value)	Cox’s hazard regression		
				Hazardratio	95%CI	*p*-value
T status	T1–2	35	0.028*	1.227	0.398–3.780	0.721
	T3–4	15				
N status	N0	32	0.139	0.142	0.042–0.478	0.002*
	N1–3	18				
CRP	≥0.1	24	0.080	1.178	0.596–2.326	0.637
	<0.1	26				
PNI	≥50	26	0.099	0.786	0.452–1.368	0.394
	<50	24				
serum IL-8	≥7 pg/ml	32	0.079	1.071	0.538–2.131	0.846
	<7 pg/ml	18				
IL-8(T)	+	24	0.007*	0.274	0.084–0.889	0.031*
	−	26				
CD163(IF)	High	25	0.002*	2.625	1.312–5.253	0.006*
	Low	25				

PNI: Prognostic Nutritional Index.

*denotes *p*<0.05, indicating statistically significance.

The multivariate analysis was performed next. In the Cox’s hazard regression test, significant correlations of N status, IL-8(T) and CD163(IF) with DSF was observed (*P* = 0.002, *P* = 0.031 and *P* = 0.006, respectively) (Table 4). These three factors may thus be independent factors affecting the DFS of OSCC patients.

Although 50 patients may not be enough for the most accurate analysis, we believe that the current data shows this study to be worth for being extended to larger patient groups.

### Combinations of independent factors for predicting clinical outcome of the OSCC patients

**Figure 3:**
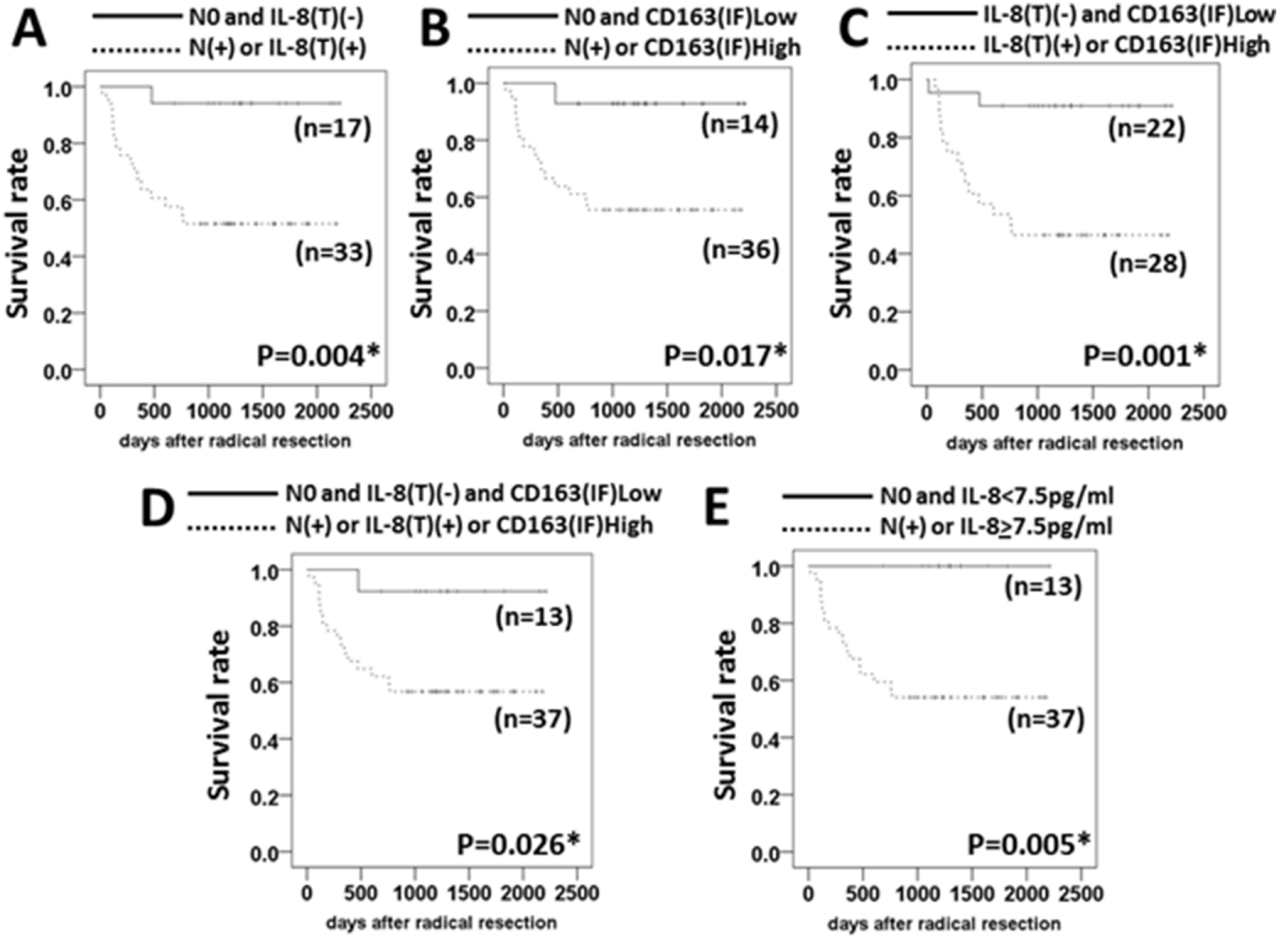
The combination of independent factors for predicting the DFS of OSCC patients. Differences in the DFS of OSCC patients with (A) N0 and IL-8(T)(−) vs. N(+) or IL-8(T)(+), (B) N0 and CD163(IF)Low vs. N(+) or CD163(IF)High, (C) IL-8(T)(−) and CD163(IF)Low vs. IL-8(T)(+) or CD163(IF)High, (D) N0 and IL-8(T)(−) and CD163(IF)Low vs. N(+) or IL-8(T)(+) or CD163(IF)High, (E) N0 and IL-8<7 pg/ml vs. N(+) or IL-8≥7 pg/ml were calculated by log-rank test. *P<0.05.

More useful biomarker(s) for predicting the clinical outcome of the resectable OSCC patients may be established by combining independent factors. We found that the DFSs were significantly longer in the patients with N0 and IL-8(T)(−), with N0 and CD163(IF)Low, with IL-8(T)(−) and CD163(IF)Low, and with N0 and IL-8(T)(−) and CD163(IF)Low compared to those with N(+) or IL-8(T)(+), with N(+) or CD163(IF)High, with IL-8(T)(+) or CD163(IF) high, and with N(+) or IL-8(T)(+) or CD163(IF)High (*P* = 0.004, *P* = 0.017, *P* = 0.001 and *P* = 0.026, respectively) (Fig. 3A–D).

Although the difference in DFS between the patients with IL-8(T)(−) and CD163(IF)Low and those with IL-8(T)(+) or CD163(IF)High showed the best p-value (*P* = 0.001), there were two patients in the IL-8(T)(−) and CD163(IF)Low group who showed relapse events. Only one relapse event was observed in other group with low relapse risk (Fig. 3A−D).

Interestingly, there was a marked and significant difference in DFS between the patients with N0 and low serum IL-8 and those with N(+) or high serum IL-8 (*P* = 0.005) (Fig. 3E), and all of the patients with N0 plus low levels of serum IL-8 have shown no relapse event as of this writing.

### Induction of CD163-positive M2 macrophages by IL-8

**Figure 4:**
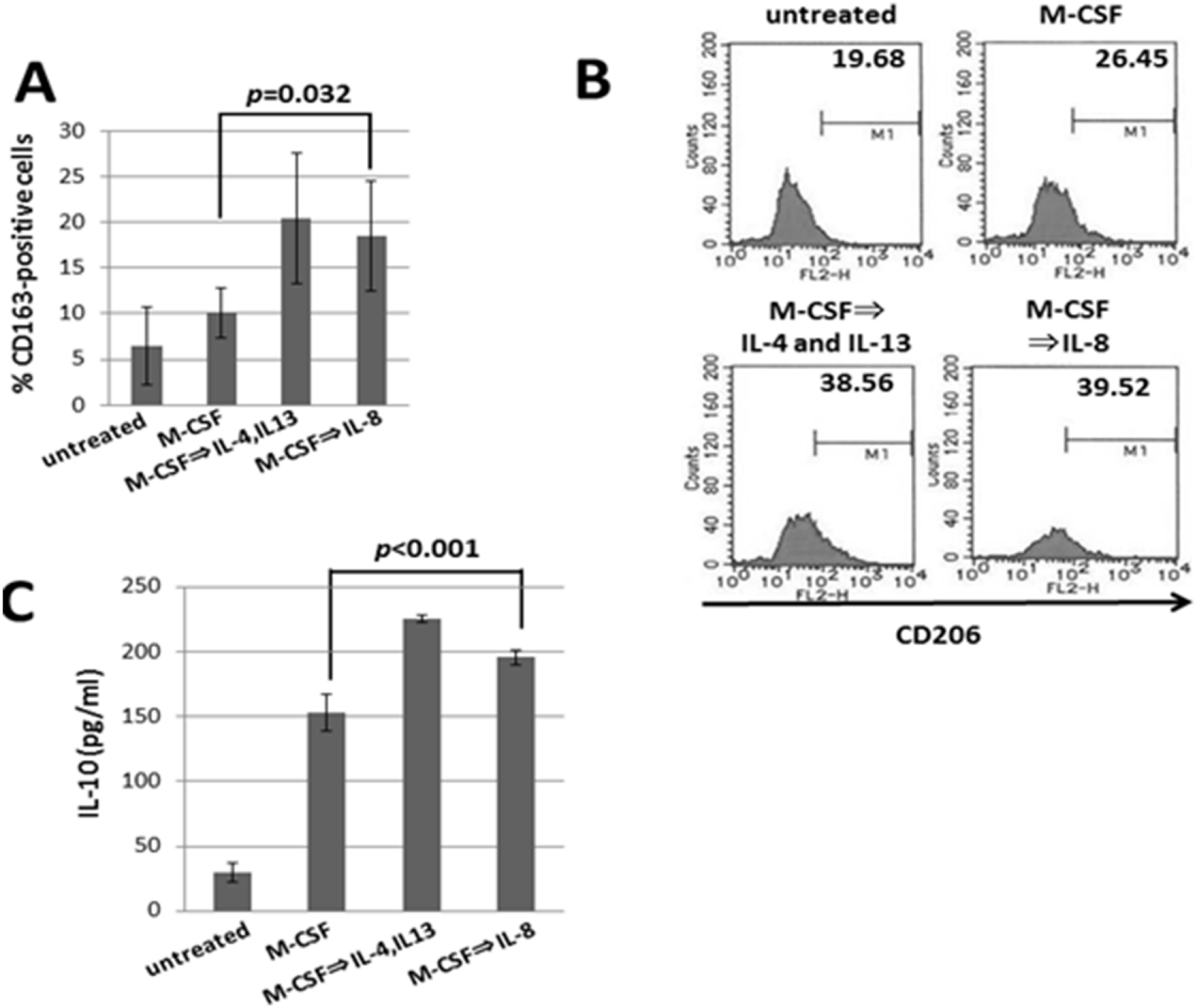
Generation of CD163-positive M2 macrophages by IL-8. Healthy donor-derived monocytes were treated with M-CSF (25 ng/ml) for 5 days and then IL-4 (20 ng/ml) and IL-13 (20 ng/ml) for 2 days, or with M-CSF (25 ng/ml) for 5 days and then IL-8 (10 ng/ml) for 2 days. The cell-surface expression of CD163 (A) and CD206 (B) of the cells was evaluated by using a flow cytometric analysis and IL-10 in the culture supernatants was measured by ELISA (R&D Systems) (C). Bars denote SD of 5 samples.

Based on the results from the study using patients’ specimens, we conducted the in vitro experiments to examine whether IL-8 may induce CD163-positive M2 macrophages from human PBMC-derived adherent cells. Results showed that IL-8 statistically significantly enhanced the number of the CD163-positive cells induced by M-CSF (Fig. 4A). In addition, we have also evaluated the induction of the cells positive for CD206 which is another marker for M2 macrophages. We repeated the experiments independently 4 times, and the similar results were obtained. Representative data have been shown in Fig. 4B. IL-8 augmented the number of CD206-positive cells which were induced by M-CSF. Because the values of the leucocyte subset are generally different in a baseline by each independent donor, statistical analysis is difficult to complete. Significant difference was obtained in CD163-positive cell number, whereas was not obtained in CD206. Although Both CD163 and CD206 are the markers of M2 macrophage, there may be some difference in an expression pattern. Furthermore, it has been also indicated that IL-8 significantly increased the production of IL-10 (Fig. 4C).

These results strongly suggested that IL-8 may cause a poor clinical outcome in OSCC patients via enhancing the generation of M2 macrophages which can produce immune-suppressive cytokines such as IL-10.

## Discussion

Factor(s) that can be detected by a peripheral blood examination are potential biomarker candidate(s) for predicting therapeutic effects and patients’ prognoses because it is technically easy to measure such factors, without a significant burden on the patients. In addition, such biomarker(s) may be used for patients with unresectable tumors since they can be obtained using only peripheral blood, not surgical specimen. The findings from the present study indicate that a patient’s serum IL-8 level may reflect his or her tumor microenvironment, which shows the expression of IL-8 in cancer cells and the infiltration of CD163-positive macrophages into the tumor invasive front. The serum IL-8 level may also be a useful biomarker at least in patients with early-stage (Stage I/II) OSCC. The DFS rate is 100% in early-stage OSCC patients with low levels of serum IL-8. Adjuvant and/or neo-adjuvant therapies may be necessary for patients with high levels of serum IL-8, even if they have early-stage OSCC. Our present findings also strongly suggest that IL-8 expression and the infiltration of CD163-positive M2 macrophages in the tumor microenvironment might be biomarkers for affecting and for predicting the clinical outcome of patients with any stage of OSCC, including advanced OSCC (Stage III/IV). Our statistical analyses revealed that there was a significant and strong difference in the DFS between the patients who showed N0 and low serum IL-8 and those who showed N(+) or high serum IL-8 (*P* = 0.005). No relapse event has occurred in the patients with N0 plus low levels of serum IL-8. The combination of N status with the circulating IL-8 level may be a new criterion for discriminating high-risk and low-risk patients with resectable OSCC. In addition, the results of the present multivariate analysis indicate that N status, IL-8 expression in the tumor and the infiltration of CD163-positive macrophages are independent factors which can affect and predict the clinical outcome of OSCC patients. Studies with larger numbers of patients are necessary to determine which combination is the most useful biomarker for OSCC patients, and a multicenter study toward this end is now being conducted.

As shown in Table 3, serum IL-8 was significantly correlated with IL-8(T) and with CD163(IF) in all stage patients (P = 0.033 and P = 0.038) as well as in Stage I/II patients (P = 0.018 and P = 0.021). IL-8(T) and CD163(IF) was also significantly correlated in all stage patients (P = 0.0003). Most of Stage III/IV patients (18 of 23 patients) showed high serum IL-8, and the tendency that serum IL-8 levels in Stage III/IV patients were higher than in Stage I/II patients was observed (P = 0.077). Whereas, no correlation between clinical stage and IL-8(T) as well as CD163(IF) (P = 0.773 and P = 1.000). Therefore, serum IL-8 might not be correlated with IL-8(T) as well as with CD163(IF) in Stage III/IV patients (P = 0.621 and P = 0.640).

In the present in vitro experiments, IL-8 induced CD163-positive M2 macrophages producing IL-10. This is the first report which shows direct induction of M2 macrophages by IL-8 although it is known that M2 macrophages secrete IL-8 [18], [19]. It is possible that IL-8 produced by cancer cells leads to poor clinical outcomes of patients with OSCC via the generation and activation of M2 macrophages. It has been reported that IL-8 and VEGF secreted by the alternatively activated macrophages accelerate tumor expansion via angiogenesis [20]. The immunohistochemical staining of the same specimens as those used in the present study revealed that the number of CD34-positive endothelial cells in the tumor tissues was significantly correlated with poor clinical outcomes of the OSCC patients; however, there was no significant correlation between the CD34-positive cell number and IL-8 expression or CD163-positive M2 macrophage-infiltration in the tumor tissues (authors’ personal communications). Thus, IL-8 and CD163-positive macrophages might elicit tumor relapse and/or post-operative cervical LN metastasis via any other mechanisms besides tumor angiogenesis (e.g., the suppression of antitumor immunity) in the tumor microenvironment.

In the present study, Foxp3-positive cell infiltration in the tumor tissue did not correlate with the patients’ survival or with other immunological parameters such as serum IL-8, IL-8(T) and CD163(IF). Foxp3-positive T cells are conventionally thought to suppress antitumor immunity, resulting in poor clinical outcomes in cancer patients. However, several recent reports demonstrated that cancer patients with high levels of tumor-infiltrating Foxp3-positive cells showed favorable clinical outcomes [21], and that anti-inflammatory cytokines (e.g., IL-10 and transforming growth factor-beta [TGF-β]) produced by Foxp3+ Tregs suppress IL-6, IL-8 and TNF-α [22] which may accelerate tumor progression. The role of Foxp3-positive cells in the clinical outcome of cancer patients is remains controversial.

Since the present findings also strongly suggest that IL-8 is not only a prognostic marker but also a factor that may contribute to a poor prognosis, the agents that can block the activity of IL-8 may be useful for improving the clinical outcome of patients with high IL-8 levels. We are now preparing a clinical trial for OSCC patients using IL-8 inhibitors including a humanized anti-human IL-8 monoclonal antibody [23] and some nutritional supplements that can suppress the upstream signals of IL-8 production, e.g. NF-κB and STAT3 [24], [25]. We expect that the ongoing prospective study will elucidate the prognostic and predictive significance of IL-8 reflecting the tumor microenvironment with the infiltration of CD163-positive M2 macrophages, and that it will be possible to conduct a clinical trial of an IL-8 inhibitor for high-risk OSCC patients.
